# Supplementary material for: Nanosecond Pulsed Electric Fields Induce Endoplasmic Reticulum Stress Accompanied by Immunogenic Cell Death in Murine Models of Lymphoma and Colorectal Cancer
Source: Cancers (Basel). 2019 Dec 17;11(12):2034. doi: 10.3390/cancers11122034 (PMC6966635; doi:10.3390/cancers11122034)
Supplement: Supplementary file 1 [file cancers-11-02034-s001.pdf]

Supplementary Materials: Nanosecond Pulsed Electric Fields Induce Endoplasmic Reticulum Stress Accompanied by Immunogenic Cell Death in Murine Models of Lymphoma and Colorectal Cancer

Alessandra Rossi, Olga N. Pakhomova, Peter A. Mollica, Maura Casciola, Uma Mangalanathan, Andrei G. Pakhomov and Claudia Muratori

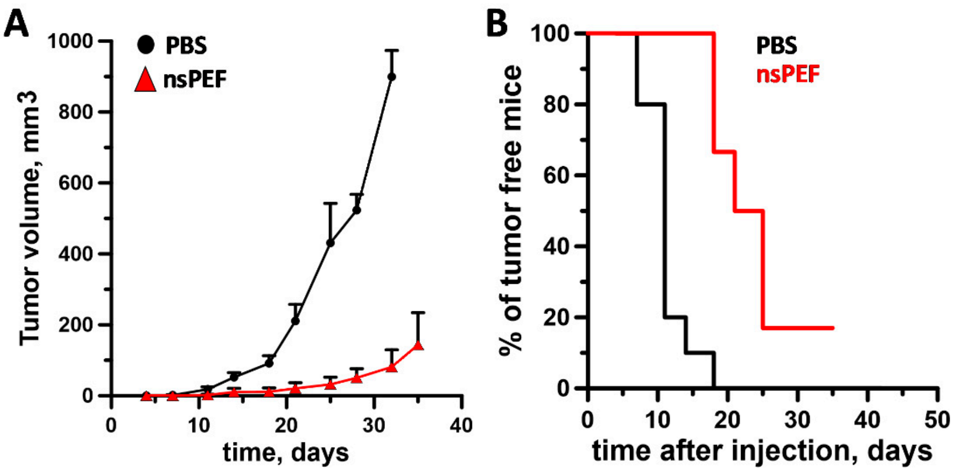

Figure S1. CT26 tumor growth curves (**A**) and % of tumor free animals (**B**) in mice that developed tumor at the vaccination site. CT26 tumor cells were treated with nsPEF (600, 200 ns, 7 kV/cm, 10 Hz) and immediately injected into the flank of syngeneic mice (0.6×10^6 cells/mouse). Control groups were vaccinated with PBS. After 7 days, animals were challenged with live cells (0.1×10^6 cells/mouse) into the opposite flank. Mean +/− s.e., $n = 10, 6$ for PBS, and nsPEF groups, respectively.

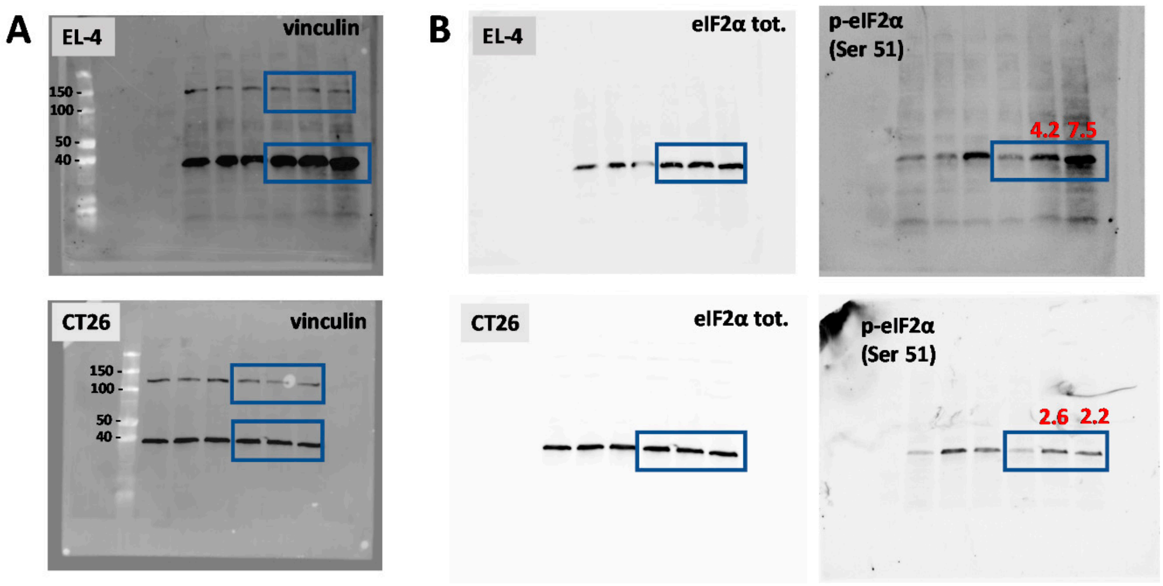

Figure S2. Full-length immunoblots images for figure 1. For both EL-4 (top panels) and CT26 (bottom panels) membranes were incubated with an anti p-eIF2*α*, stripped, incubated with an anti-eIF2*α* to measure the total amount of the protein, and finally incubated with an anti-vinculin antibody. To show specific band sizes in (**A**) vinculin and marker images were overlaid. Because membranes were not stripped in between eIF2*α* tot. and vinculin antibody incubations, images show both vinculin (140

kDa) and eIF2α (37 kDa) signals. (**B**) Full-length images for eIF2α tot and p-eIF2α. Numbers in red are quantifications of p-eIF2α expressed as fold to sham.

© 2019 by the authors. Licensee MDPI, Basel, Switzerland. This article is an open access article distributed under the terms and conditions of the Creative Commons Attribution (CC BY) license (http://creativecommons.org/licenses/by/4.0/).
